# Supplementary material for: Transcriptional regulation of endothelial cell behavior during sprouting angiogenesis
Source: Nat Commun. 2017 Sep 28;8:726. doi: 10.1038/s41467-017-00738-7 (PMC5620061; doi:10.1038/s41467-017-00738-7)
Supplement: Supplementary file 1 — Supplementary Information [file 41467_2017_738_MOESM1_ESM.pdf]

a

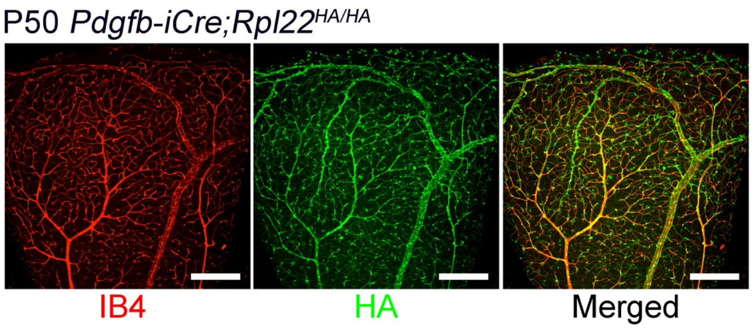

b

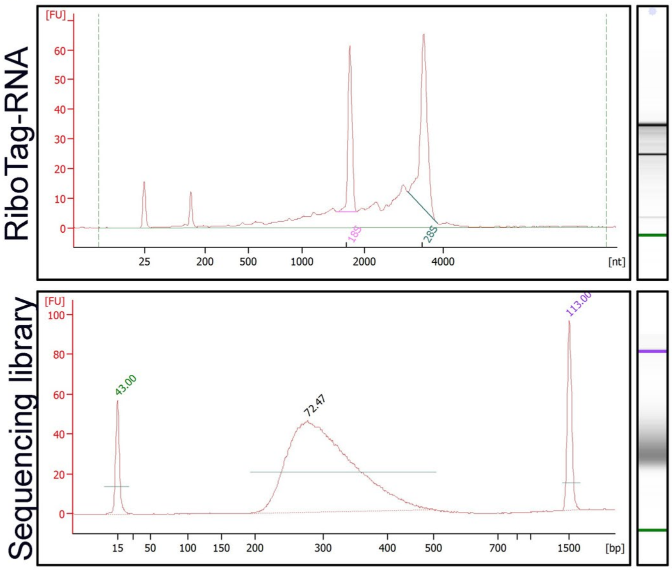

	P6	P10	P15	P21	P50
RNA (ng)	17.79	45.57	14.20	15.59	5.38
	15.86	31.98	15.05	14.84	11.05
	16.55	27.42	33.76	9.68	12.03
**Average**	**16.73**	**34.99**	**21.00**	**13.37**	**9.49**
RIN	7.80	6.90	8.00	8.10	7.60
	8.20	7.20	8.30	8.00	7.50
	8.10	7.20	6.80	8.00	7.40
**Average**	**8.03**	**7.10**	**7.70**	**8.03**	**7.50**

c

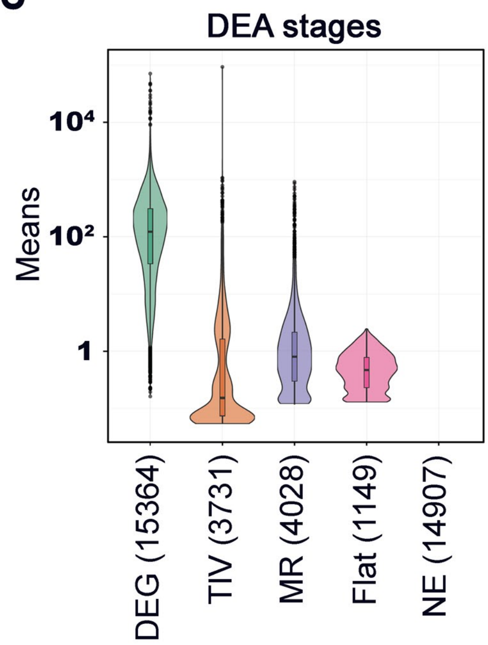

Supplementary Figure 1. DEA of retinal EC profiling during postnatal development

(a) P50 *Pdgfb-iCre Rpl22*$^{HA/HA}$ retina stained with Isolectin B4 (IB4, red) and anti-HA antibody (green). Scale bars represent 300 μm.

(b) Bioanalyzer analysis of retinal EC-RiboTag RNA and sequencing library. Lower table shows the total amount of immunoprecipitated RNA and RNA integrity number (RIN) for each sample.

(c) Retinal EC transcriptome analysis. NE, no expression; Flat, Flat profiles (T.fit); MR, non-significant after model refinement (p.vector); TIV, time-independent variability (get.siggenes); DEG, differentially expressed genes.

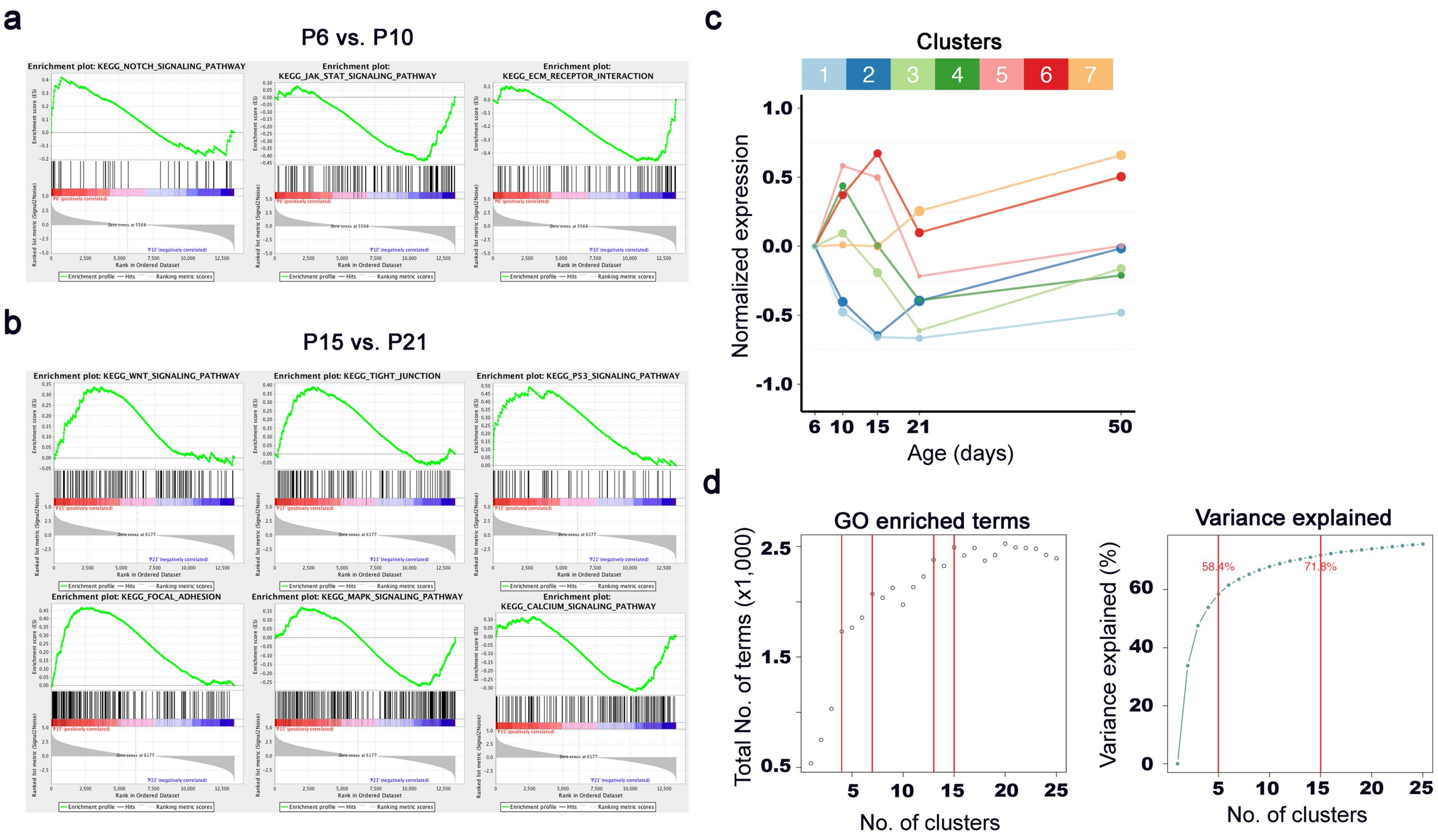
a
P6 vs. P10
Enrichment plot: KEGG_NOTCH_SIGNALING_PATHWAY
Enrichment plot: KEGG_JAK_STAT_SIGNALING_PATHWAY
Enrichment plot: KEGG_ECM_RECEPTOR_INTERACTION
'P6' (positively correlated)
Zero cross at 5568
'P10' (negatively correlated)
Rank in Ordered Dataset
Enrichment profile
Hits
Ranking metric scores
Enrichment score (ES)
Ranked list metric (Signal2Noise)
b
P15 vs. P21
Enrichment plot: KEGG_WNT_SIGNALING_PATHWAY
Enrichment plot: KEGG_TIGHT_JUNCTION
Enrichment plot: KEGG_P53_SIGNALING_PATHWAY
Enrichment plot: KEGG_FOCAL_ADHESION
Enrichment plot: KEGG_MAPK_SIGNALING_PATHWAY
Enrichment plot: KEGG_CALCIUM_SIGNALING_PATHWAY
'P15' (positively correlated)
Zero cross at 6177
'P21' (negatively correlated)
c
Clusters
1
2
3
4
5
6
7
Normalized expression
Age (days)
d
GO enriched terms
Total No. of terms (x1,000)
No. of clusters
Variance explained
Variance explained (%)
58.4%
71.8%
No. of clusters

Supplementary Figure 2. GSEA of retinal EC DEGs

(a and b) Enrichment plots of GSEA results in early (P6) vs. mid (P10) stages (a), or mid (P15) vs. late (P21) stages (b). Curated gene sets derived from the KEGG pathway database (c2.cp.kegg.v5.1.symbols.gmt) were used. Only some representative examples are shown.

(c) Expression dynamics of 7 DEG clusters.

(d) Total number of enriched GO terms (top) and percentage of variance explained (bottom) according to the number of clusters in the k-means clustering algorithm.

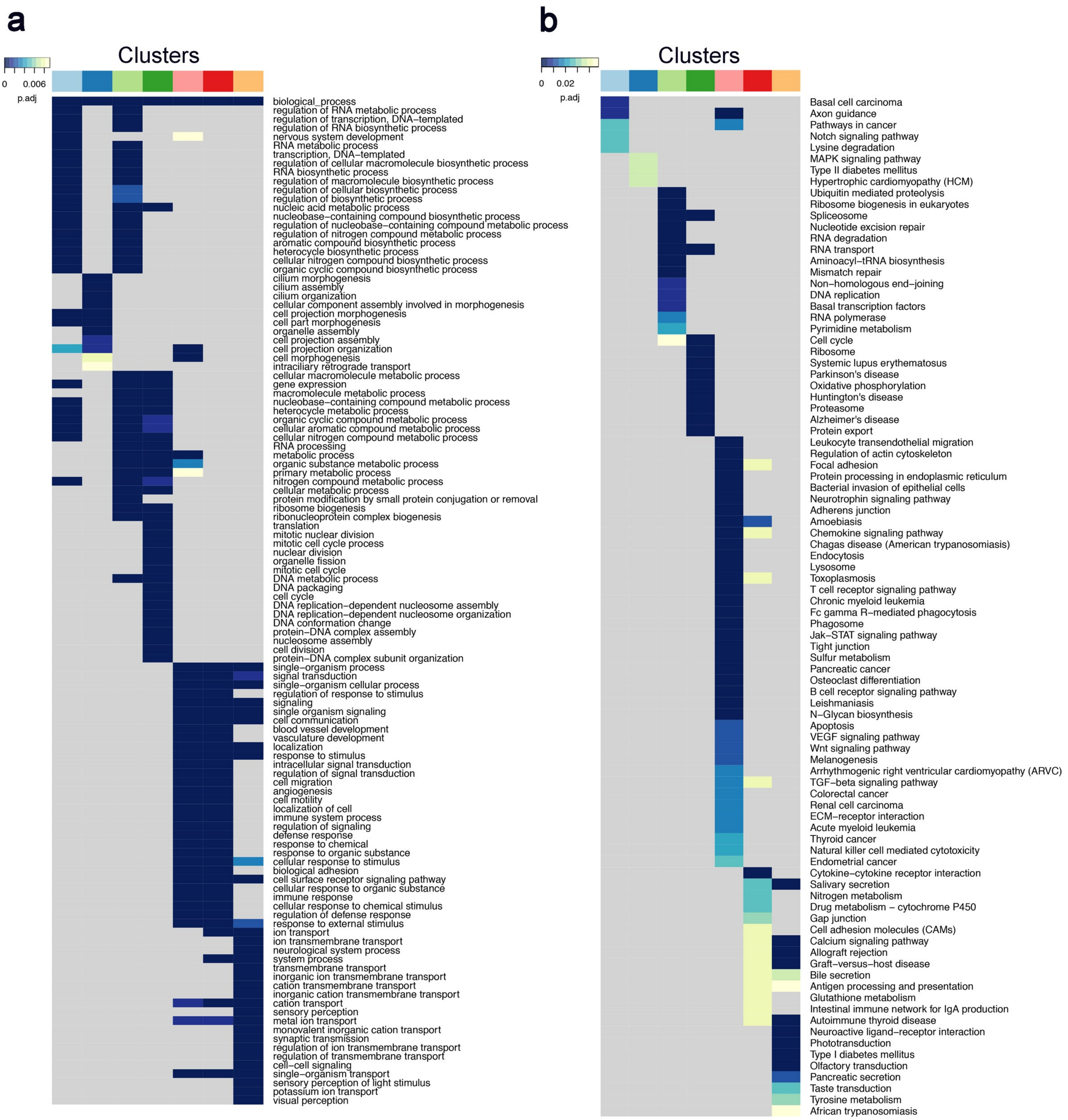

a
Clusters
0
0.006
p.adj
biological_process
regulation of RNA metabolic process
regulation of transcription, DNA−templated
regulation of RNA biosynthetic process
nervous system development
RNA metabolic process
transcription, DNA−templated
regulation of cellular macromolecule biosynthetic process
RNA biosynthetic process
regulation of macromolecule biosynthetic process
regulation of cellular biosynthetic process
regulation of biosynthetic process
nucleic acid metabolic process
nucleobase−containing compound biosynthetic process
regulation of nucleobase−containing compound metabolic process
regulation of nitrogen compound metabolic process
aromatic compound biosynthetic process
heterocycle biosynthetic process
cellular nitrogen compound biosynthetic process
organic cyclic compound biosynthetic process
cilium morphogenesis
cilium assembly
cilium organization
cellular component assembly involved in morphogenesis
cell projection morphogenesis
cell part morphogenesis
organelle assembly
cell projection assembly
cell projection organization
cell morphogenesis
intraciliary retrograde transport
cellular macromolecule metabolic process
gene expression
macromolecule metabolic process
nucleobase−containing compound metabolic process
heterocycle metabolic process
organic cyclic compound metabolic process
cellular aromatic compound metabolic process
cellular nitrogen compound metabolic process
RNA processing
metabolic process
organic substance metabolic process
primary metabolic process
nitrogen compound metabolic process
cellular metabolic process
protein modification by small protein conjugation or removal
ribosome biogenesis
ribonucleoprotein complex biogenesis
translation
mitotic nuclear division
mitotic cell cycle process
nuclear division
organelle fission
mitotic cell cycle
DNA metabolic process
DNA packaging
cell cycle
DNA replication−dependent nucleosome assembly
DNA replication−dependent nucleosome organization
DNA conformation change
protein−DNA complex assembly
nucleosome assembly
cell division
protein−DNA complex subunit organization
single−organism process
signal transduction
single−organism cellular process
regulation of response to stimulus
signaling
single organism signaling
cell communication
blood vessel development
vasculature development
localization
response to stimulus
intracellular signal transduction
regulation of signal transduction
cell migration
angiogenesis
cell motility
localization of cell
immune system process
regulation of signaling
defense response
response to chemical
response to organic substance
cellular response to stimulus
biological adhesion
cell surface receptor signaling pathway
cellular response to organic substance
immune response
cellular response to chemical stimulus
regulation of defense response
response to external stimulus
ion transport
ion transmembrane transport
neurological system process
system process
transmembrane transport
inorganic ion transmembrane transport
cation transmembrane transport
inorganic cation transmembrane transport
cation transport
sensory perception
metal ion transport
monovalent inorganic cation transport
synaptic transmission
regulation of ion transmembrane transport
regulation of transmembrane transport
cell−cell signaling
single−organism transport
sensory perception of light stimulus
potassium ion transport
visual perception
b
Clusters
0
0.02
p.adj
Basal cell carcinoma
Axon guidance
Pathways in cancer
Notch signaling pathway
Lysine degradation
MAPK signaling pathway
Type II diabetes mellitus
Hypertrophic cardiomyopathy (HCM)
Ubiquitin mediated proteolysis
Ribosome biogenesis in eukaryotes
Spliceosome
Nucleotide excision repair
RNA degradation
RNA transport
Aminoacyl−tRNA biosynthesis
Mismatch repair
Non−homologous end−joining
DNA replication
Basal transcription factors
RNA polymerase
Pyrimidine metabolism
Cell cycle
Ribosome
Systemic lupus erythematosus
Parkinson's disease
Oxidative phosphorylation
Huntington's disease
Proteasome
Alzheimer's disease
Protein export
Leukocyte transendothelial migration
Regulation of actin cytoskeleton
Focal adhesion
Protein processing in endoplasmic reticulum
Bacterial invasion of epithelial cells
Neurotrophin signaling pathway
Adherens junction
Amoebiasis
Chemokine signaling pathway
Chagas disease (American trypanosomiasis)
Endocytosis
Lysosome
Toxoplasmosis
T cell receptor signaling pathway
Chronic myeloid leukemia
Fc gamma R−mediated phagocytosis
Phagosome
Jak−STAT signaling pathway
Tight junction
Sulfur metabolism
Pancreatic cancer
Osteoclast differentiation
B cell receptor signaling pathway
Leishmaniasis
N−Glycan biosynthesis
Apoptosis
VEGF signaling pathway
Wnt signaling pathway
Melanogenesis
Arrhythmogenic right ventricular cardiomyopathy (ARVC)
TGF−beta signaling pathway
Colorectal cancer
Renal cell carcinoma
ECM−receptor interaction
Acute myeloid leukemia
Thyroid cancer
Natural killer cell mediated cytotoxicity
Endometrial cancer
Cytokine−cytokine receptor interaction
Salivary secretion
Nitrogen metabolism
Drug metabolism − cytochrome P450
Gap junction
Cell adhesion molecules (CAMs)
Calcium signaling pathway
Allograft rejection
Graft−versus−host disease
Bile secretion
Antigen processing and presentation
Glutathione metabolism
Intestinal immune network for IgA production
Autoimmune thyroid disease
Neuroactive ligand−receptor interaction
Phototransduction
Type I diabetes mellitus
Olfactory transduction
Pancreatic secretion
Taste transduction
Tyrosine metabolism
African trypanosomiasis

Supplementary Figure 3. Top enriched GO terms per clusters

(a and b) Heat map of retinal EC DEG clusters showing distinctive gene enrichment of cluster-specific biological processes. Top 20% of GO terms (a) and top 40% of KEGG reactome signaling pathway (b) with FDR-adjusted P value < 0.01 are shown.

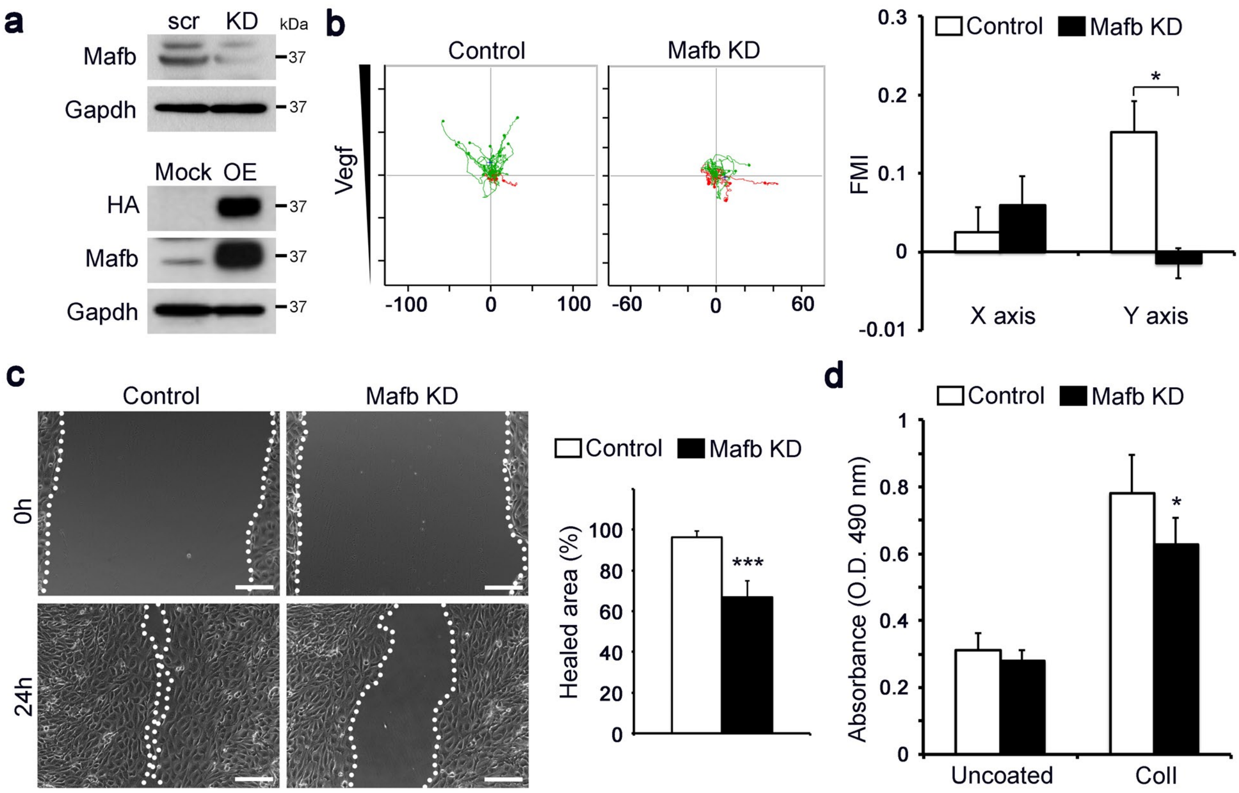

a
scr
KD
kDa
Mafb
Gapdh
37
Mock
OE
HA
Mafb
Gapdh
b
Control
Mafb KD
Vegf
-100
0
100
-60
0
60
Control
Mafb KD
FMI
0.3
0.2
0.1
0
-0.01
X axis
Y axis
*
c
Control
Mafb KD
0h
24h
Healed area (%)
100
80
60
40
20
0
***
d
Control
Mafb KD
Absorbance (O.D. 490 nm)
1
0.8
0.6
0.4
0.2
0
Uncoated
CollI
*

Supplementary Figure 4. Role of MafB in ECs in vitro

(a) Western blot of endogenous MafB or HA-tagged form of MafB proteins (36 kDa) in MS1 ECs infected with lentivirus containing scrambled shRNA (scr), *Mafb*-targeting shRNA (KD, knockdown), no-insert (Mock), or HA-tagged *Mafb* cDNA (OE, overexpression). Gapdh was used as a loading control.

(b) Cell Migration tracks (left) and forward migration index (right) of Control and *Mafb* KD MS1 ECs in the presence of a VEGF-A concentration gradient. The initial point for each cell is the intersection between the X- and Y-axes and the source of VEGF is at the top. X- and Y-axes are perpendicular and parallel to the VEGF gradient, respectively. n = 36, Error bars represent mean ± s.e.m., *P < 0.05.

(c) Scratch wound healing assay with Control and *Mafb* KD MS1 ECs. Scale bars represent 100 μm. The area of wound at 0 hour and 24 hours were measured. n = 8, Error bars represent mean ± s.e.m., ***P < 0.0001.

(d) Cell adhesion assay with Control and *Mafb* KD MS1 ECs at 30 minutes after cell seeding to uncoated or Collagen I-coated (ColI) wells. n = 3, Error bars represent mean ± s.e.m., *P < 0.05.

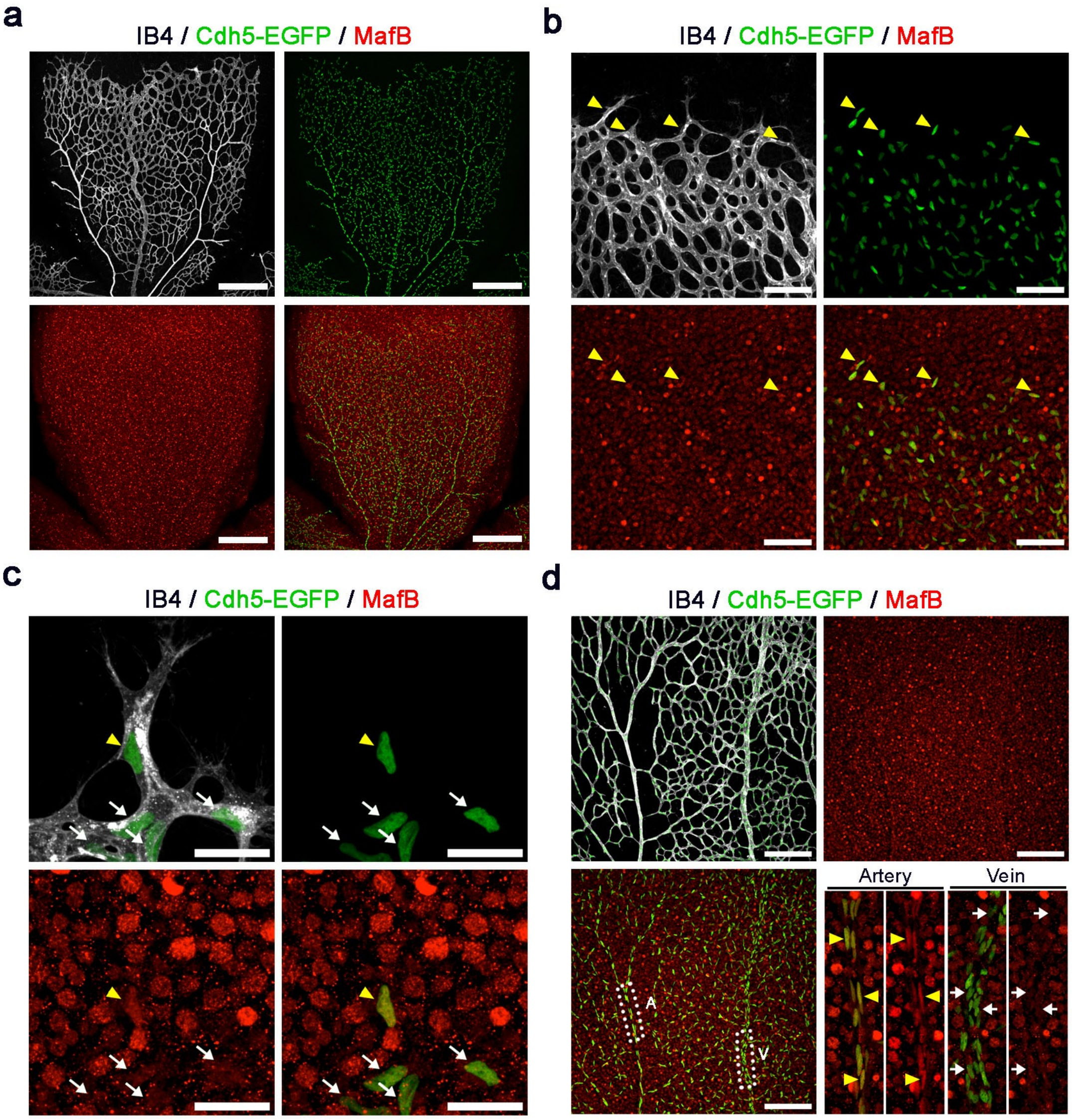
a
IB4 / Cdh5-EGFP / MafB
b
IB4 / Cdh5-EGFP / MafB
c
IB4 / Cdh5-EGFP / MafB
d
IB4 / Cdh5-EGFP / MafB
A
V
Artery
Vein

Supplementary Figure 5. Expression of MafB in P6 mouse retina

(a-d) P6 *Cdh5-EGFP* transgenic mouse retinas stained with IB4 (white), anti-EGFP (green) and anti-MafB (red). Yellow arrowheads indicate enriched expression of MafB in tip cells (b and c) or arterial ECs (d). White arrows indicate lower expression of MafB in stalk/plexus ECs (b and c) or venous ECs (d). Scale bars represent 300 μm (a), 75 μm (b), 30 μm (c) and 150 μm (d).

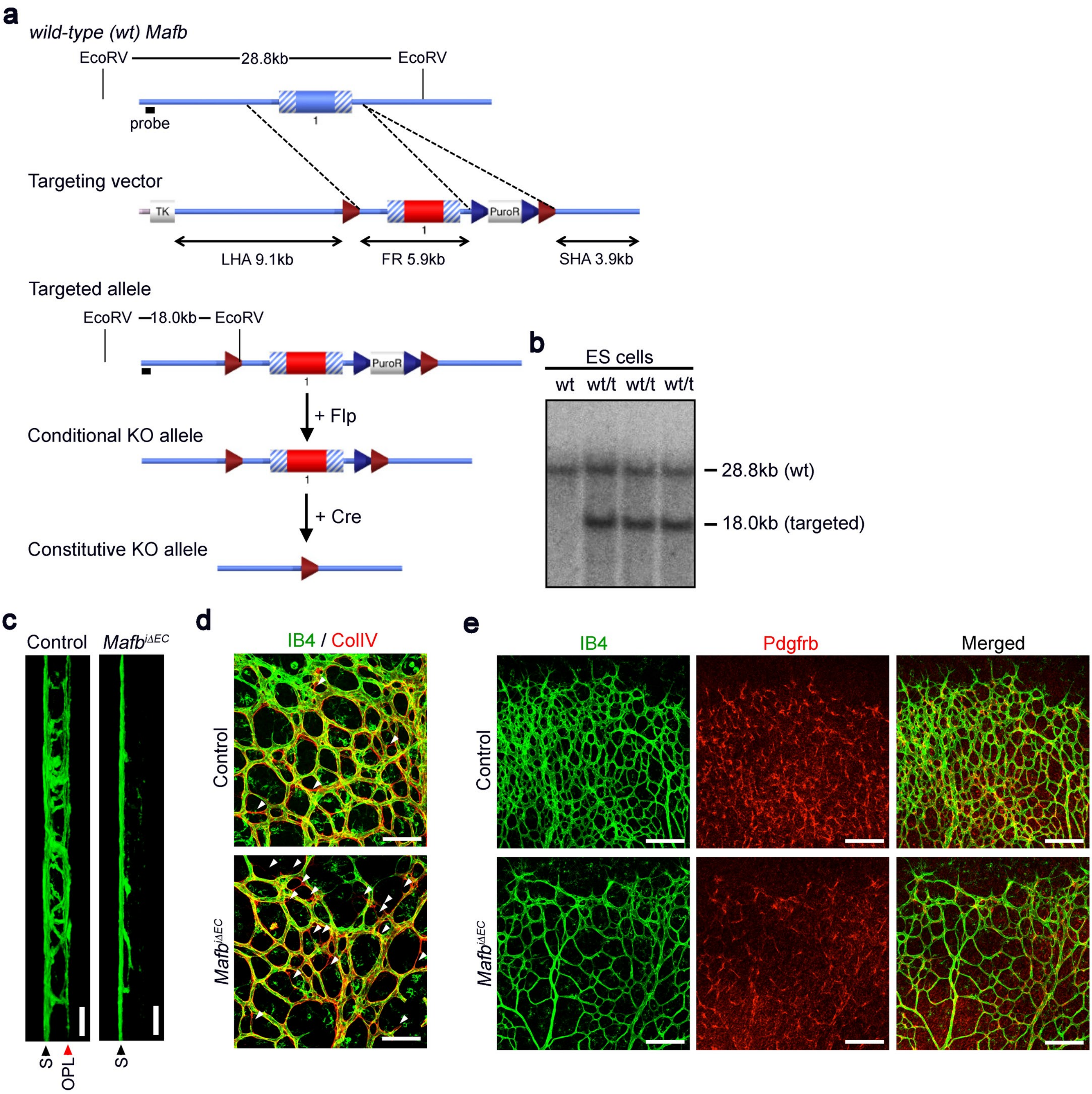
a
wild-type (wt) Mafb
EcoRV
28.8kb
EcoRV
probe
Targeting vector
TK
PuroR
LHA 9.1kb
FR 5.9kb
SHA 3.9kb
Targeted allele
EcoRV
18.0kb
EcoRV
PuroR
+ Flp
Conditional KO allele
+ Cre
Constitutive KO allele
b
ES cells
wt
wt/t
wt/t
wt/t
28.8kb (wt)
18.0kb (targeted)
c
Control
Mafb iΔEC
S
OPL
S
d
IB4 / CollV
Control
Mafb iΔEC
e
IB4
Pdgfrb
Merged
Control
Mafb iΔEC

Supplementary Figure 6. Characterization of MafB function in ECs

(a) Structure of the wild-type (wt) *Mafb* locus, the targeting vector, the targeted recombinant allele (t), the conditional KO allele, and the constitutive KO allele resulting from *Mafb* inactivation. The loxP and FRT recombination sites (brown and blue triangles, respectively), the EcoRV restriction sites and probe for Southern blot analysis are indicated. SHA, short homology arm; LHA, long homology arm; FR, loxP-flanked region.

(b) Southern blot analysis of the wild-type (wt) ES cells and of the 3 targeted (wt/t) clones used to establish independent recombinant mouse lines. EcoRV-restricted DNA yielded 28.8- and 10.0-kb bands for the wild-type and recombinant alleles, respectively, with the probe in b.

(c) IB4 staining of retinas from *Mafb*$^{i\Delta EC}$ and control littermates (Control) at P10. Y-Z orthogonal confocal plane images are shown. S, superficial plexus; OPL, outer plexiform layer. Scale bars represent 40 μm.

(d) Staining of P6 control or *Mafb*$^{i\Delta EC}$ retinas with IB4 (green) and anti-ColIV antibody (red). Arrowheads indicate IB4-negative and ColIV-positive empty matrix sleeves. Scale bars represent 75 μm.

(e) Staining of P6 control or *Mafb*$^{i\Delta EC}$ retinas with IB4 (green) and anti-PDGFRβ antibody (red). Scale bars represent 150 μm.

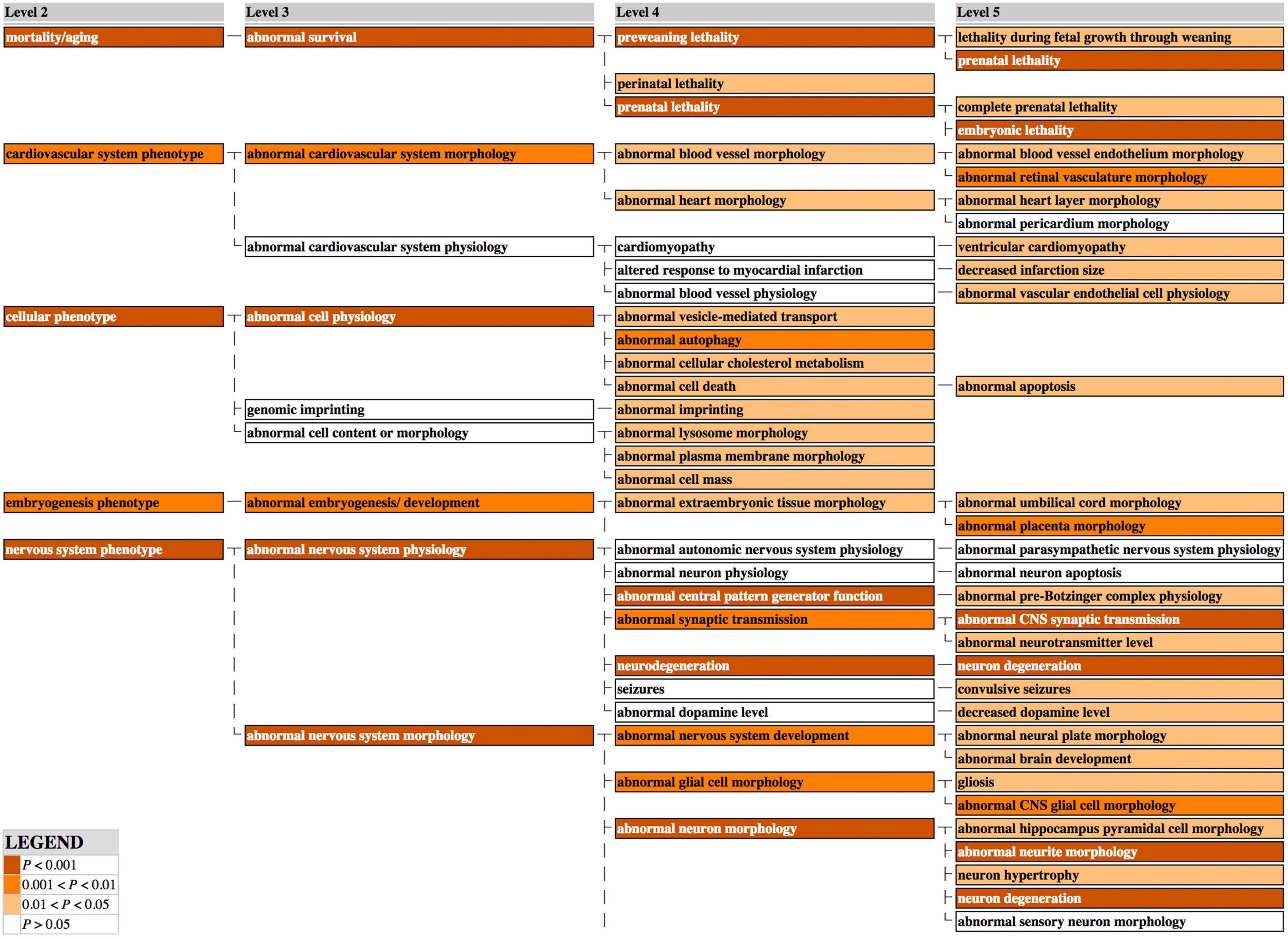

Level 2
Level 3
Level 4
Level 5
mortality/aging
abnormal survival
preweaning lethality
lethality during fetal growth through weaning
prenatal lethality
perinatal lethality
prenatal lethality
complete prenatal lethality
embryonic lethality
cardiovascular system phenotype
abnormal cardiovascular system morphology
abnormal blood vessel morphology
abnormal blood vessel endothelium morphology
abnormal retinal vasculature morphology
abnormal heart morphology
abnormal heart layer morphology
abnormal pericardium morphology
abnormal cardiovascular system physiology
cardiomyopathy
ventricular cardiomyopathy
altered response to myocardial infarction
decreased infarction size
abnormal blood vessel physiology
abnormal vascular endothelial cell physiology
cellular phenotype
abnormal cell physiology
abnormal vesicle-mediated transport
abnormal autophagy
abnormal cellular cholesterol metabolism
abnormal cell death
abnormal apoptosis
genomic imprinting
abnormal imprinting
abnormal cell content or morphology
abnormal lysosome morphology
abnormal plasma membrane morphology
abnormal cell mass
embryogenesis phenotype
abnormal embryogenesis/ development
abnormal extraembryonic tissue morphology
abnormal umbilical cord morphology
abnormal placenta morphology
nervous system phenotype
abnormal nervous system physiology
abnormal autonomic nervous system physiology
abnormal parasympathetic nervous system physiology
abnormal neuron physiology
abnormal neuron apoptosis
abnormal central pattern generator function
abnormal pre-Botzinger complex physiology
abnormal synaptic transmission
abnormal CNS synaptic transmission
abnormal neurotransmitter level
neurodegeneration
neuron degeneration
seizures
convulsive seizures
abnormal dopamine level
decreased dopamine level
abnormal nervous system morphology
abnormal nervous system development
abnormal neural plate morphology
abnormal brain development
abnormal glial cell morphology
gliosis
abnormal CNS glial cell morphology
abnormal neuron morphology
abnormal hippocampus pyramidal cell morphology
abnormal neurite morphology
neuron hypertrophy
neuron degeneration
abnormal sensory neuron morphology
LEGEND
P < 0.001
0.001 < P < 0.01
0.01 < P < 0.05
P > 0.05

Supplementary Figure 7. MamPhEA result of P6 *Mafb*$^{i\Delta EC}$ retinal ECs

MamPhEA performed with 'Fisher's exact test - differentially enriched (two sided)' and 'use loss-of-function phenotypes only' options. Results are displayed in hierarchical structure of phenotype ontology with MGI pre-defined phenotypes at level 2 to level 5.

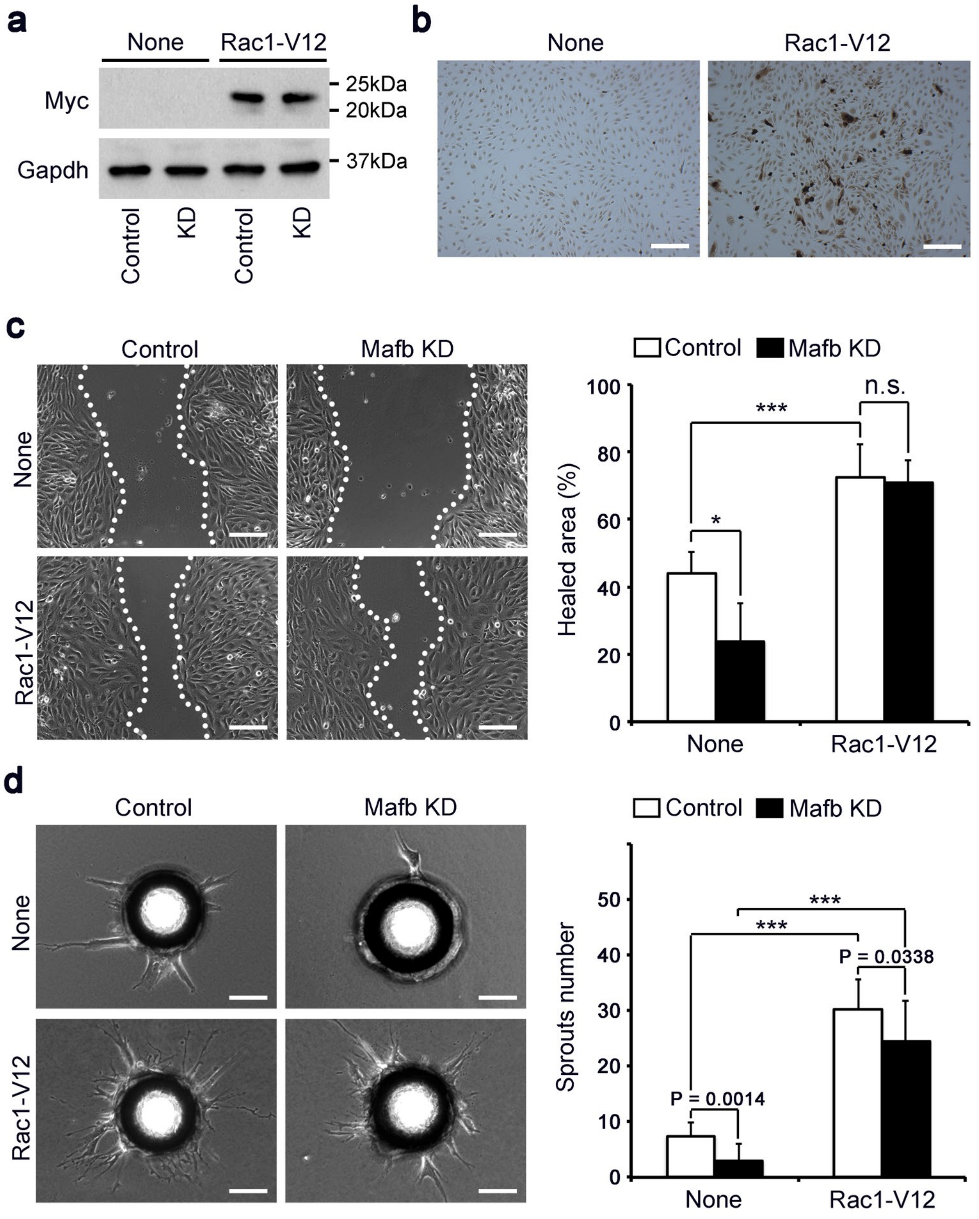

a
None
Rac1-V12
Myc
Gapdh
25kDa
20kDa
37kDa
Control
KD
Control
KD
b
None
Rac1-V12
c
Control
Mafb KD
None
Rac1-V12
Control
Mafb KD
Healed area (%)
***
n.s.
*
0
20
40
60
80
100
None
Rac1-V12
d
Control
Mafb KD
None
Rac1-V12
Control
Mafb KD
Sprouts number
***
***
P = 0.0338
P = 0.0014
0
10
20
30
40
50
None
Rac1-V12

Supplementary Figure 8. Constitutive activation of Rac1 rescues MafB KD cell migration defects

(a) Western blot of Myc-tagged Rac1-V12 (22 kDa) in Control or MafB-KD MS1 ECs. Gapdh was used as a loading control.

(b) Immunostaining of Myc tag in MS1 cells treated with only PEI (None) or transfected with Rac1-V12 construct. Scale bar, 200 μm.

(c) Scratch wound healing assay with Control and *Mafb* KD MS1 ECs transfected with Rac1-V12 construct. Wound areas at 12 hours after incubation are shown. Graph on the right shows closure of scratch wounds at 12 hours by Control and *Mafb* KD MS1 ECs with or without Rac1-V12 expression, respectively. Scale bars represent 100 μm. n = 8, Error bars represent mean ± s.e.m., *P < 0.05, ***P < 0.0001, n.s ; not significant.

(d) 3-dimensional fibrin gel bead sprouting assay with Control and *Mafb* KD MS1 ECs with or without Rac1-V12 transfection. Representative spheroids are shown for each condition at culture day 1. Scale bars represent 50 μm. Average numbers of sprouts per spheroid in each condition are shown on the right. n = 12, Error bars represent mean + s.d., ***P < 0.0001.

Unprocessed original scans of blots

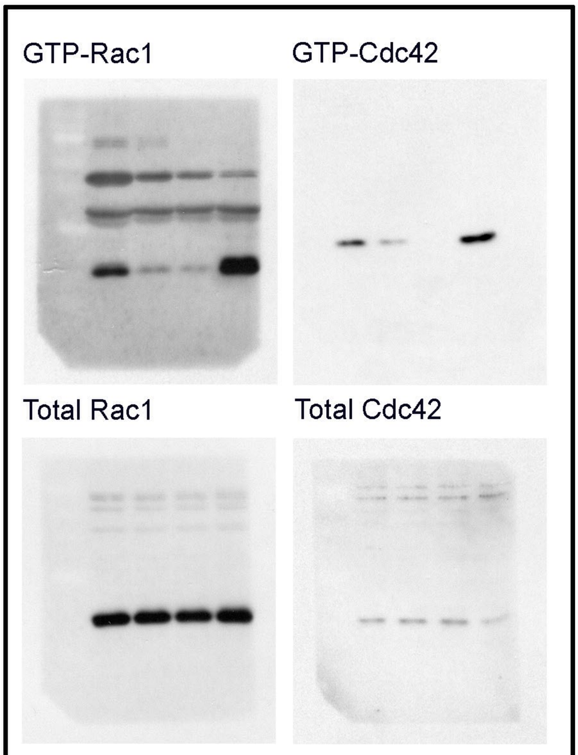

Figure 7f

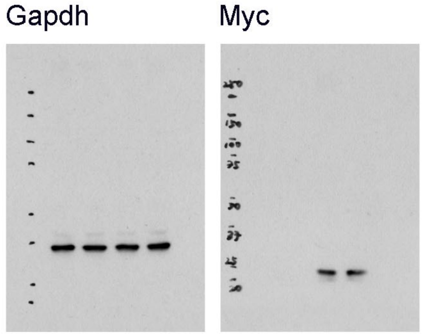

Supplementary Figure 8a

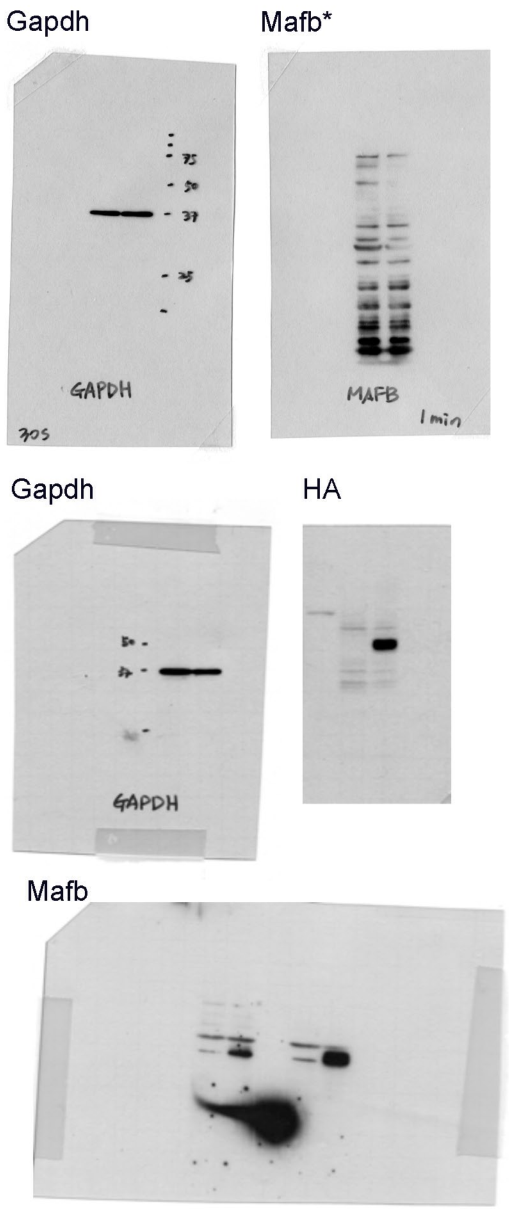

Supplementary Figure 4a

* Due to the low expression level of endogenous MafB, this blot was developed with ECL Prime Western blotting detection reagent (GE Healthcare; RPN2232).
The rest of blots were developed with ECL Western blotting detection reagent (GE Healthcare; RPN2209).

Supplementary Figure 9. Scans of unprocessed Western blots

Unprocessed images of western blot scanning shown in Fig. 7f, Supplementary Fig. 4a, and Supplementary Fig. 8a.

Retinal EC-RiboTag-RNA-Seq reads usage

Sample	Sequenced pairs	Mapped pairs	%	Multiple alignment	%
P6-1_S1	7,929,279	7,314,243	92.24%	458,192	5.78%
P6-2_S2	7,726,301	7,204,729	93.25%	421,098	5.45%
P6-3_S3	9,394,635	8,694,924	92.55%	492,308	5.24%
P10-1_S1	8,972,269	8,104,083	90.32%	461,592	5.14%
P10-2_S2	9,346,850	8,492,580	90.86%	535,922	5.73%
P10-3_S3	9,660,498	8,792,248	91.01%	495,798	5.13%
P15-1_S1	8,821,866	7,789,667	88.30%	395,168	4.48%
P15-2_S2	10,560,733	9,231,974	87.42%	443,354	4.20%
P15-3_S3	9,800,344	8,316,744	84.86%	426,908	4.36%
P21-1_S1	7,919,292	7,406,975	93.53%	532,956	6.73%
P21-2_S2	7,386,185	6,844,035	92.66%	414,366	5.61%
P21-3_S3	7,093,142	6,635,217	93.54%	457,938	6.46%
P50-1_S1	8,407,899	7,787,986	92.63%	384,018	4.57%
P50-2_S2	8,494,139	7,808,742	91.93%	392,052	4.62%
P50-3_S3	9,006,215	8,282,996	91.97%	411,622	4.57%
**Average**	**8,701,310**	**7,913,810**	**91.14%**	**448,219**	**5.20%**

Supplementary Table 1. RNA-Seq reads usage

Tophat2 alignment statistics for paired-end reads of retinal EC RiboTag-RNA-Seq libraries.
