# Supplementary material for: Transcriptional regulation of endothelial cell behavior during sprouting angiogenesis
Source: Nat Commun. 2017 Sep 28;8:726. doi: 10.1038/s41467-017-00738-7 (PMC5620061; doi:10.1038/s41467-017-00738-7)
Supplement: Supplementary file 3 — Description of Additional Supplementary Files [file 41467_2017_738_MOESM3_ESM.pdf]

Description of Additional Supplementary Files

File Name: Supplementary Data 1
Description: **FPKM Table of Differentially expressed genes in Clusters** Fragments per kilobase of exon per million reads mapped (FPKM) of all differentially expressed genes in retinal ECs during postnatal development.

File Name: Supplementary Data 2
Description: **SMARA-identified Putative Key TFs** List of putative key TFs identified by ISMARA. Results from comparison between two sequential developmental stages (P6 vs. P10, P10 vs. P15, P15 vs. P21, and P21 vs. P50) and across all 5 stages are shown.

File Name: Supplementary Data 3
Description: **Differentially expressed genes in Mafb cKO retinal ECs** Differentially expressed genes in retinal ECs of $Mafb^{i\Delta EC}$ in compared with control retinal ECs at P6. Upregulated and down-regulated genes in Mafb mutant mice are shown in different sheets.
